# The Impact of Hepatic Cirrhosis on Chronic Obstructive Pulmonary Disease in the United States: A Nationwide Analysis

**DOI:** 10.7759/cureus.16368

**Published:** 2021-07-13

**Authors:** James R Pellegrini, Rezwan Munshi, Pranavi Patel, Brandon Pelletier, Palakkumar Patel, Paul Mustacchia

**Affiliations:** 1 Internal Medicine, Nassau University Medical Center, East Meadow, USA; 2 Gastroenterology and Hepatology, Nassau University Medical Center, East Meadow, USA

**Keywords:** hepatic cirrhosis, chronic obstructive pulmonary disease, hospital readmission rate, hepatology, pulmonology, healthcare utilization

## Abstract

We aimed to study the impact of Hepatic Cirrhosis (HC) on chronic obstructive pulmonary disease (COPD).

Our study is a retrospective cohort study using the 2016-2017 National Readmission Database (NRD). NRD is part of the Healthcare Cost and Utilization Project (HCUP), organized and supported by means of the Agency for Healthcare Research and Quality (AHRQ). Patients were included if they were 18 years or older and had a principal diagnosis of COPD based on International Classification of Diseases, Tenth Revision (ICD-10- CM) codes and had a secondary diagnosis of HC.

A total of 505,004 patients were included in the study with a diagnosis of COPD, 6196 (1.23%) of whom had HC. HC was found to be more common amongst male patients between the ages of 50 and 65 years. Medicare beneficiaries with high comorbidity burden, lower socioeconomic status, and those who received treatment in a large urban teaching hospital also had higher rates of HC. Patients with HC and COPD correlated to an increase of in-hospital mortality (adjusted odds ratio (aOR: 2.21, p<0.001) and 30-day hospital readmission rate (aOR: 1.23, p<0.001) compared with patients without HC. The in-hospital mortality rate was higher during readmission compared with index admissions (5.01% versus 2.16%; p<0.001). In addition, HC was associated with higher morbidity including prolonged mechanical ventilation (aOR: 1.39, p<0.001), resource utilization with prolong length of stay (LOS) (adjusted mean difference (aMD: 0.51, p<0.001), higher total hospitalization charges (aMD: 4967, p<0.001), and costs (aMD: 1200, p<0.001). Both patient groups had similar odds of being intubated (aOR: 1.18, p-0.13), tracheostomy (aOR: 0.81, p-069) and bronchoscopy rates (aOR: 1.27, p-0.36). The most common causes of hospital readmission were found to be COPD with acute exacerbation (19.7%), sepsis, unspecified organism (6.0%, acute and chronic respiratory failure with hypoxia (4.2%), acute on chronic systolic heart failure (3.9%), and hepatic failure, unspecified coma (3.1%). Various autonomous prognosticators of readmission were sex (particularly female), alcohol dependence, LOS greater than 7 days, lower comorbidity burden, and discharge to short term hospital or against medical advice. On the other hand, males, patients without a history of alcohol dependence, greater comorbidity burden, and LOS fewer than 3 days, were less likely to be readmitted.

We found that HC is related to higher in-hospital mortality, LOS, increased mechanical ventilation, resource utilization with prolonged LOS, hospital costs, odds of intubation, and tracheostomy and bronchoscopy rates. Our study aims to shed light on the impact of HC on COPD in hopes to improve future management.

## Introduction

Hepatic cirrhosis (HC) and chronic obstructive pulmonary disease (COPD) are two of the most well-known medical diseases in the world today. The correlation between chronic liver disease and decreased lung function was noted by clinicians as early as 1884, with clubbing and cyanosis observed in cirrhotic patients [1]. While this correlation has long been known to exist, its consequences continue to be explored and merit further examination [2]. Our study demonstrates the degree to which HC exacerbates the outcomes of patients with COPD, as evidenced by mortality rates, readmission rates, measures of morbidity and burden to the healthcare system.

## Materials and methods

Data source

This is a retrospective cohort study using the 2016-2017 National Readmission Database (NRD). NRD is part of the Healthcare Cost and Utilization Project (HCUP), organized and supported by means of the Agency for Healthcare Research and Quality (AHRQ). The NRD is one of the largest all-payer publicly available healthcare databases inside the United States. It encompasses information of both weighted and unweighted healthcare facility encounters every year. The weighted information allows for measuring country wide estimates. IRB approval was not required for this study as information in the database was de-identified and made publicly available for research purposes. 

Study population

Patients were included if they were 18 years or older and had a principal diagnosis of COPD based on International Classification of Diseases, Tenth Revision (ICD-10- CM) codes and had a secondary diagnosis of HC. The primary outcome was in-hospital mortality. Secondary outcomes were 30-day readmission rates, morbidity, resource utilization, and predictors of readmission. Exclusion criteria for this study was age under 18 years, non-elective admission, and discharge in December. 

Statistical analysis

Multivariate linear/logistic and cox regression models were used to adjust for confounders and identify predictors of readmission. Trends in readmission of COPD were analyzed by the linear-by-linear association test. A two-step hierarchical multivariate regression model was used, after adjusting for comorbidities, to measure hospitalization outcomes secondary to HC in COPD patients. Stata Version 16.1 by StataCorp LLC (College Station, TX) was utilized for all statistical analyses.

## Results

A total of 505,004 patients were included in the study with a diagnosis of COPD, 6196 (1.23%) of whom had HC. HC was found to be more common amongst male patients between the ages of 50 and 65 years (Table 1). Medicare beneficiaries with high comorbidity burden, lower socioeconomic status, and those who received treatment in a large urban teaching hospital also had higher rates of HC (Table 1).

**Table 1:** Baseline Patient Characteristics

Variable	COPD without HC, n(%)	COPD with HC, n(%)	P Value
Total N- 505,004	N-498,809	N-6,195	
Gender			<0.001
Male	41.9	54.3	
Female	58.1	45.7	
Mean Age	67	63	<0.001
Age group			<0.001
18-30 year	9.9e-04	0	
30- 40 year	0.8	0.3	
40-50 year	4.8	5.8	
50-65 year	36.7	58.1	
>65 year	57.6	35.7	
Length of stay			<0.001
< 3 days	33.5	29.3	
3 - 7 days	51.8	50.9	
>7 days	14.7	19.8	
Insurance provider			<0.001
Medicare	72.3	63.26	
Medicaid	14.2	26.2	
Private	10.7	8.0	
Uninsured	2.8	2.6	
Charlson Comorbidity Index			<0.001
1	36.2	0.2	
2	25.8	25.7	
3 or More	37.9	74.1	
Median income in patient Zip code			0.01
$1-$38,999	38.3	41.1	
$39,000-$47,999	27.8	27.6	
$48,000-62,999	21.2	20.5	
>$63,000	12.8	10.9	
Patient Residence			<0.001
Large Metropolitan areas with at least 1 million residents	49.1	52.7	
Small metropolitan areas with less than 1 million residents	34.7	36.1	
Micropolitan areas	10.8	8.1	
Not metropolitan or micropolitan (nonurban residual)	5.5	3.1	
Hospital Size			0.001
Small	20.9	17.5	
Medium	30.2	29.6	
Large	50.0	52.9	
Hospital teaching Status			<0.001
Non-Teaching	49.0	39.9	
Teaching	51.0	60.1	
Hospital Location			<0.001
Rural	16.3	11.2	
Urban	83.7	88.8	
Hospital Volume quintile			0.001
1(lowest)	1.5	0.7	
2	5.7	5.0	
3	12.2	12.0	
4	23.2	21.7	
5(Highest)	57.5	60.6	
Discharge type			<0.001
Routine	66.0	61.74	
Transfer to short term hospital	4.9	0.7	
Other transfer, including skilled nursing facility, intermediate care, other facility	11.3	12.07	
Home Health Care	19.1	19.9	
Against medical	2.0	3.4	
Died in hospital	1.1	2.2	
Discharged alive, destination unknown	1.1e-04	2.7e-04	
Treatment level Outcomes			
Intubation			<0.001
No	98.31	97.3	
Yes	1.7	2.8	
Prolonged Mechanical ventilation			<0.001
No	99.4	98.7	
Yes	0.6	1.3	
Chest tube Placement			0.34
No	99.8	99.7	
Yes	0.2	0.3	
Tracheostomy			0.25
No	99.9	99.8	
Yes	8.1e-04	0.14	
Bronchoscopes			0.06
No	99.7	99.6	
Yes	0.3	0.4	

Patients with HC and COPD correlated to an increase of in-hospital mortality (adjusted odds ratio (aOR: 2.21, p<0.001) and 30-day hospital readmission rate (aOR: 1.23, p<0.001) compared with patients without HC (Table 2). The in-hospital mortality rate was higher during readmission compared with index admissions (5.01% versus 2.16%; p<0.001) (Table 2). In addition, HC was associated with higher morbidity including prolonged mechanical ventilation (aOR: 1.39, p<0.001), resource utilization with prolonged length of stay (LOS) (adjusted mean difference (aMD: 0.51, p<0.001), higher total hospitalization charges (aMD: 4967, p<0.001), and costs (aMD: 1200, p<0.001) (Table 1). Both patient groups had similar odds of being intubated (aOR: 1.18, p-0.13), tracheostomy (aOR: 0.81, p-069) and bronchoscopy rates (aOR: 1.27, p-0.36). The most common causes of hospital readmission were found to be; HC with COPD with acute exacerbation (19.7%), sepsis, unspecified organism (6.0%, acute and chronic respiratory failure with hypoxia (4.2%), acute on chronic systolic heart failure (3.9%), and hepatic failure, unspecified coma (3.1%) (Table 3). Various autonomous prognosticators of readmission were sex (particularly female), alcohol dependence, LOS greater than 7 days, lower comorbidity burden, and discharge to short term hospital or against medical advice. On the other hand, males, patients without a history of alcohol dependence, greater comorbidity burden, and LOS fewer than 3 days, were less likely to be readmitted (Table 4). 

**Table 2:** Respiratory outcomes of Patients with COPD with and without Hepatic Cirrhosis

Outcomes	COPD with Hepatic Cirrhosis	COPD without Hepatic cirrhosis	P value
In-Hospital mortality rates	2.16%	1.07%	<0.001
30-day Mortality rates	3.15%	1.58%	<0.001
Readmission Mortality rates	5.01%	3.74%	0.01
Readmission rates	24.41%	16.35%	<0.001

**Table 3:** Most common causes of readmission for patients with COPD and Hepatic Cirrhosis

Cause of readmission	Percentage of patients
COPD with Acute Exacerbation	19.7%
Sepsis, Unspecified organism	6.0%
Acute and Chronic Respiratory Failure with Hypoxia	4.2%
Acute on Chronic systolic heart failure	3.9%
Hepatic Failure, Unspecified coma	3.1%

**Table 4:** Independent predictor of 30-day readmission

Variable	Adjusted Hazard Ratio(95% Confidence Interval)	P value
Age group		
30 to 40 year	1.93 ( 0.84 – 4.43)	0.13
40 to 50 year	1.13 ( 0.76 – 1.61)	0.52
50 to 65 year	1.00 (0.81 – 1.23)	0.98
>65 year	Reference	Reference
Length of stay		
< 3 days	Reference	Reference
3 - 7 days	1.02 (0.84 – 1.23)	0.85
>7 days	1.29 ( 1.01 – 1.65)	0.04
Gender		
Male	Reference	Reference
Female	1.21 (1.03- 1.42)	0.01
Alcohol Dependence		
No	Reference	Reference
Yes	1.20 ( 1.01- 1.42 )	0.03
Insurance Provider		
Medicare	Reference	Reference
Medicaid	1.22 (0.98 – 1.47 )	0.07
Private	0.98 ( 0.71 – 1.37)	0.93
Uninsured	0.75 ( 0.37 – 1.42)	0.406
Charlson Comorbidity Score		
<1	Reference	Reference
2	0.33 (0.15- 0.73)	0.006
>3	0.48( 0.22 – 1.63 )	0.06
Discharge		
Routine	Reference	Reference
Transfer to short term hospital	3.92 (2.05 – 7.52 )	<0.001
Other transfer, including skilled nursing facility, intermediate care, other facility	1.04 (0.79 – 1.37)	0.75
Home Health Care	1.18 (0.96 – 1.45 )	0.11
Against medical	2.12 ( 1.45 – 3.09 )	<0.001

## Discussion

Hepatopulmonary syndrome (HPS) and portopulmonary hypertension (POPH) are two known lung complications of liver disease that could be contributing to the increased frequency and severity of COPD observed in HC patients [2]. Additionally, alcohol and cigarette smoking are independently known to have considerable deleterious effects on both the liver and lung tissues and may also underlie the worse outcomes observed in COPD patients with HC [3,4,5].

HPS is defined as hypoxemia secondary to liver disease (portal hypertension or cirrhosis) [1]. In 1966, Berthelot et al. were the first to demonstrate the vascular pathology in patients with HC characterized by precapillary arteriole vasodilation with conspicuously absent changes to the alveoli and connective tissues of the lung [6]. The pathophysiology of HPS in humans has not been fully elucidated; however, multiple mechanisms have been implicated. One such mechanism involves increased circulating nitrous oxide (NO) levels, as HPS patients have been observed to exhale greater levels of NO than non-HPS patients, with these differences rectified by liver transplant [7,8]. However, therapies targeting the elevated NO levels have had mixed results, implicating pulmonary vasculature remodeling that occurs over a longer time frame. The dilation of capillaries and precapillary arterioles in HPS cause derangements in hypoxia-induced vasoconstriction, leading to ventilation-perfusion (V/Q) mismatch, right to left shunting, and subsequent hypoxemia [9]. It is reasonable to conclude that V/Q mismatch from HPS may be a contributing factor to the higher frequency of COPD observed in HC patients.

POPH is defined as elevated pressures in the pulmonary circulation with right ventricular dysfunction resulting from increased pulmonary vascular resistance. POPH is believed to be another potential sequelae of liver disease and has been documented in 1-2% of patients with portal hypertension [10]. The pathophysiology of POPH is thought to be multifactorial, with decreased clearance of vasoactive substances like endothelin and serotonin produced in the splanchnic circulation being implicated. Histologically, findings are similar to other pulmonary arterial hypertension causes, with arterial wall thickening and fibrosis that occlude the vessel lumens [11]. Interestingly, the severity of liver disease and portal hypertension have not been shown to be related to the severity of portopulmonary HTN, and whereas HPS tends to resolve with liver transplant, POPH tends to persist [10].

Other commonly known disruptors of liver and lung function include cigarette smoke with alcohol use. In this study, alcohol dependence was shown to be an independent risk factor for readmission (Table 4). While its hepatic toxicity is well recognized, alcohol has also been shown to affect the lungs. In fact, patients with alcohol use disorder are more susceptible to developing pneumonia, ARDS, and tuberculosis [5]. On the other hand, while it was not a variable examined in this study, cigarette smoking is known to cause COPD and liver dysfunction. It acts on the lung and liver through direct or indirect toxin effects, immunological, and oncogenic effects [3]. Tobacco and alcohol use are closely related; tobacco-dependent subjects have been found to have over 4 times the likelihood for alcohol dependence than the general population, while those with alcohol dependence have been found to have 3 times the likelihood for tobacco dependence [12]. Moreover, concurrent dependence on both tobacco and alcohol have been shown to increase risk for heart, liver, and lung disease more than either behavior in isolation [13]. Thus, comorbid tobacco and alcohol use may also be contributing to the worse outcomes observed in COPD patients with HC. Due to the modifiable nature of smoking status and alcohol, modulating use of both risk factors is an important area for meaningful intervention. The relationship between smoking and alcohol use and their consequences in patients with COPD and HC is an area for further investigation.

It is of particular importance to note that the most common cause of readmission for patients with COPD and HC was found in patients who developed acute COPD exacerbation, sepsis, and acute on chronic heart failure exacerbation (Table 3). In order to improve readmission rates, patients with COPD should be adequately monitored to ensure they are taking the appropriate medication regimen for their COPD. This regimen should include a long acting muscarinic antagonist (LAMA) (i.e., tiotropium), a long acting beta-2-agonist (LABA) (i.e., salmeterol) and an inhaled corticosteroid (ICS) (i.e. Budesonide) [12]. This “triple therapy” of LAMA + LABA + ICS has been shown to decrease exacerbations and hospitalizations in patients with COPD [12]. In order to prevent infections in patients with COPD and HC, they should be given yearly influenza vaccine, the pneumococcal vaccine twice after the age of 65, the Hepatitis A vaccine, and the Hepatitis B vaccine [12,13]. Improvement in readmission rates caused by acute on chronic heart failure exacerbation, more commonly seen in HC patients, can be achieved by optimizing heart failure medication regimen. Liver transplantation is often curative for HC but has several contraindications such as advanced cardio-pulmonary disease which may be more profound in patients with COPD and HC [13]. 

The significant increase in risk of morbidity and mortality illuminate the additional burden of COPD with concurring HC, and the need to better manage and monitor this patient population. Increased length of stay and increased readmissions place a high burden on valuable hospital resources (Tables 1 and 2). Augmented utilization of scarce hospital resources necessitates that those resources are not used elsewhere. As previously mentioned, more careful and vigorous monitoring should be considered when caring for the population of patients who have COPD with HC due to the high medical expenses associated with this combination of diseases.

## Conclusions

Overall, this study shows consequences of COPD with HC to be dire, and an area that deserves further attention from researchers and clinicians. Areas for further research include additional characterization of the consequences of concurrent liver and lung disease, the pathophysiology underlying the connection, and new treatments. Patients with COPD and HC as well as their healthcare providers should be made aware of the previously examined increase in risks to better direct preventative efforts. 
